# Volatile Composition of Sparkling Wines of cv. Chardonnay Cultivated under Different Training Systems in Serra da Mantiqueira (Brazil)

**DOI:** 10.3390/foods11111529

**Published:** 2022-05-24

**Authors:** Naíssa Prévide Bernardo, Aline de Oliveira, Renata Vieira da Mota, Francisco Mickael de Medeiros Câmara, Isabela Peregrino, Murillo de Albuquerque Regina, Eduardo Purgatto

**Affiliations:** 1 Food Sciences and Experimental Nutrition Department, School of Pharmaceutical Sciences, University of São Paulo, Avenida Prof Lineu Prestes, 580, bl 14, São Paulo 05508-000, SP, Brazil; alinedeoliveira@usp.br (A.d.O.); epurgatt@usp.br (E.P.); 2 Food Research Centre, University of São Paulo, São Paulo 05508-000, SP, Brazil; 3 Agricultural Research Centre of Minas Gerais, Experimental Farm of Caldas, Grape and Wine Technological Centre, Avenida Santa Cruz, 500, Postal Code 33, Caldas 37780-000, MG, Brazil; rvmota@epamig.br (R.V.d.M.); francisco.camara@epamig.br (F.M.d.M.C.); isabela.peregrino@yahoo.com.br (I.P.); murillo@vitaceabrasil.com.br (M.d.A.R.)

**Keywords:** crop, GDC, lyre, aroma, HS-SPME, GC-MS

## Abstract

The grapevine is a climbing plant and allows for the manipulation of vegetative canopies to change the microclimate and exposure of leaves and clusters to solar radiation, affecting the primary and secondary metabolisms of plants. Thus, this work aimed to evaluate how the lyre and Geneva double-curtain (GDC) training systems could contribute to the volatile composition of sparkling wines in replicates of vinifications carried out in the Serra da Mantiqueira (Brazil) in two consecutive summer harvests (2017 and 2018). Fifty-four free volatile compounds were identified by HS-SPME/GC-MS in the wines in both systems and vintages. Multivariate analysis differentiated the vintages in component 1 (22.7%) and the training systems in component 2 (7.1%). The crops were differentiated by aldehydes in 2017 and in 2018 by isoamyl acetate ester, probably derived from the amino acid leucine, the season having been more humid, with lower temperatures and less radiation. For the training systems, besides the alcohol compounds, the GDC was differentiated by the terpenoid compounds geranylacetone and β-damascenone, which may contribute more pleasant aromas to sparkling wines. This work promotes additional research and enables winegrowers, through the management of their vineyards, to achieve sparkling wines with different volatile compositions.

## 1. Introduction

The vine is a climbing, sarmentous plant that adapts to the most diverse training systems. Manipulating the vegetative canopy growth structure is a technique applied to define the plant’s shape. The canopy structure objectives are to expose leaf areas to maximise light absorption, optimise the leaf area-to-fruit ratios, control diseases, and avoid competition for sunlight between vines [1]. Additionally, modifying microclimatic conditions inside the canopy affects plant temperature, photosynthesis, and metabolic activity [2,3,4] and promotes good insolation and aeration of the leaves and clusters [5,6]. Some of these parameters and how they influence certain classes of volatile compounds were recently reported on by van Leeuwen et al. [7].

Sparkling wine aromas can be expressed by primary (varietal), secondary (fermentative), and tertiary (ageing on lees) compounds, which contribute to the *bouquet.* Regarding the discrimination of sparkling wines by the adopted training systems, the microclimate established in the vine due to the organisation of the sprouts and branches throughout the cycle influences the physiology of the plant and the composition of the berries, impacting wine composition and quality [8,9].

Several training systems are described in the literature and applied worldwide. In the Douro region, for the varieties Touriga Nacional and Touriga Franca, the influence of two systems on the composition of terpenes, norisoprenoids, and their precursors was evaluated, and it was shown that the double-cordon LYS 2/3 system produced grapes and Porto wines with higher contents of terpenes and norisoprenoid glycosylated fractions compared with vertical shoot positioning (VSP) [10]. In southern Italy, the interactions between soil management and training systems (bilateral and monolateral Guyot) were shown to be related significantly to aromatic compounds in Negroamaro wine [11] (and other studies). The most widely applied systems in Brazil are lyre (Y or V), VSP, GDC, and pergola systems. Papers in the literature have evaluated Y-trellis and VSP systems in the control of downy mildew and botrytis bunch rot diseases in a high-altitude region in Santa Catarina (south of Brazil) [12], the effects of VSP and Y systems on Cabernet Sauvignon grape berry ripening [13], and the agronomic performance and chemical composition differences associated with GDC, pergola, simple curtain, and VSP systems [14].

In the southeast of Brazil, the physiological responses and productivities of Syrah grapevines were assessed in different training systems [15]. However, data are lacking regarding the volatile compositions of grapes or wines under different training systems.

Serra da Mantiqueira is a new winemaking frontier for producing sparkling wines [16,17]; therefore, this study aimed to evaluate the aromatic profile of Chardonnay sparkling wines (made according to the *champenoise* method) under two training systems, lyre and GDC.

## 2. Materials and Methods

### 2.1. Experimental Design—Viticulture and Winemaking Conditions

Sparkling wines made from the Chardonnay cultivar (clone 76) grafted onto the 1103 Paulsen rootstock, trained on lyre and GDC systems with 1 m between plants, were obtained by conducting vinifications at Empresa de Pesquisa Agropecuária de Minas Gerais (EPAMIG) located in Caldas (21°55′ S and 46°23′ W), south of Minas Gerais (Brazil), in two consecutive summer vintages (2017 and 2018). This region in the Serra da Mantiqueira has an altitude of 1.100 m, loamy soil, and unirrigated vineyards.

After the must *débourbage* step, 48 h at 4 °C with the addition of pectolytic enzyme and antioxidant, the clarified musts were microvinified in 13L Pyrex bottles, to which were added hydrated *Saccharomyces bayanus* (Maurivin PDM, purchased from the Amazon Group, Brazil) and 20 g/hL activator (Actimax Vit, purchased from Agrovin, Ciudad Real, Spain) to start the first fermentation, which lasted between 15 and 20 days at 15–19 °C. Subsequently, potassium metabisulfite (50 mg/L) was added and stabilisations were performed. First, the clarification (protein stabilisation) was conducted, in two steps, one with 80 g/hL of bentonite (Laffort Microcol Alpha, purchased from Laffort, St Helena, CA, USA) and the other with 30 g/hL. A second stabilisation was performed with tartaric acid twice during 12 days at −2 °C. Thus, the stabilized base wines were obtained.

The second fermentation and ageing on lees were performed in 750 mL bottles for sparkling wines. Therefore, the *tirage liqueur* was added to the base wine, a mixture of 26 g/L of sucrose, 20 g/hL of Actimax Vit, 30 g/hL of bentonite, and 20 g/hL of Gesferm Plus (Coatec from the Amazon Group, Bento Gonçalves, RS, Brazil). After 18 months, the bottles were subjected to *remuage* in the *pupitres* for a month, followed by *degórgement* to remove the lees and add the expedition *liqueur* to each bottle (5 mL per bottle). Each 5 mL contained 85 mg of potassium metabisulfite and 0.15 g of Carboxi (Coatec, purchased from the Amazon Group, Brazil) to obtain a sparkling wine, classified as Nature, and corking.

### 2.2. Volatile Compounds Analysis

#### 2.2.1. Sample Preparation

For the volatile organic compound (VOC) analysis, the bottles of the sparkling wines were stored in an underground cellar at 16–18 °C until analysis.

At the time of analysis, the sparkling wines were refrigerated and sonicated for 2 min before extraction. Aliquots of the samples (5 mL applied for both matrices) were added to a 40 mL screw-top amber glass vial (LabSource, Cotia, SP, Brazil), then 5 mL of saturated NaCl (ACS grade, Sigma-Aldrich, St. Louis, MO, USA) solution and 50 µL of 4-methyl-2-pentanol at 1.6 mg/mL (Certified Reference Material, Sigma-Aldrich) as internal standard were also added.

#### 2.2.2. HS-SPME/GC-MS

The extraction was conducted by HS-SPME, the water bath was adjusted to 40 °C, and the vial was equilibrated for 5 min before inserting the SPME fibre, 50/30 µm 2 cm DVB/CAR/PDMS (divinylbenzene/carboxen/polydimethylsiloxane) (StableFlex, purchased from Supelco). The fibre was exposed in the headspace to capture the free volatile compounds over 50 min. The analytes absorbed in the fibre were then separated and detected using a gas chromatograph HP6890 (Series GC System G1530A) coupled to an HP model 5973 mass selective detector (Agilent Technologies, Palo Alto, CA, USA). The analytes were desorbed in the GC injection port at 250 °C, and the separation was performed through a Carbowax column (30 m × 0.25 mm × 0.25 µm). For the wines, the chromatographic ramp applied was based on Carlin et al. [18], starting at 40 °C for 5 min, ramped at 6 °C/min to 250 °C, and maintained for 10 min. The temperatures of the injector and transfer liner were maintained at 250 °C and the helium flow rate at 1.2 mL/min (analytical purity 99.999%, White Martins, São Paulo, SP, Brazil). The parameters for the mass spectrometer were set at 70 eV, 230 °C (EI source temperature) and 150 °C (quadrupole temperature). Mass spectra were acquired over *m*/*z* 30–350 using ChemStation software. The method described above was applied to the samples by evaluating the extraction conditions (bath temperature, equilibration time of the headspace, and time to capture the compounds) and, for GC-MS, the reproducibility and sensitivity parameters. A sample chromatogram is provided in the Supplementary Materials (Figure S1).

#### 2.2.3. Data Processing and Compound Assignment

Agilent MassHunter qualitative analysis software (version: B.07.00) was applied as a deconvolution tool. The compounds were assigned according to the fragmentation profile, ion abundance between the sample spectrum and the NIST library (NIST 14), and by NIST score (applied score: over 70). Additionally, the modified Kovats retention index (mKRI) was assessed to confirm the compounds by injecting saturated alkanes (C6–C30) standard 1000 µg/mL (Supelco) [19]. For the sparkling wine aroma evaluation, the compounds identified in both training systems and vintages were selected. Furthermore, the percentage of the total area detected (% TDA) was reported for the bottle replicates of each vinification for each training system and vintage.

#### 2.2.4. Statistical Analysis

MetaboAnalyst 5.0 software was applied to evaluate the clustering effects of the aromatic profile using partial least squares discriminant analysis (PLS-DA) and a loading graph of the normalised area for the compounds identified (area of the volatile compound divided by the area of the internal standard). The normalised area for each compound is provided in the Supplementary Materials (Tables S1–S4).

## 3. Results and Discussion

### 3.1. Multivariate Analysis

Replicates of vinifications were performed for the lyre and GDC systems in two consecutive summer harvests to evaluate the two stages of Chardonnay (clone 76) sparkling wine vinification by the *champenoise* method.

The two training systems evaluated in this study have a division of the vegetative canopy, being obliquely upward and vertically downward, respectively, for lyre and GDC. For both systems, the exposed leaf area is significant. However, the effect on the growth angle is associated with variations in the xylem vessels that appear to be adapted to optimize the supply of water and nutrients to the shoots [20].

**Figure 1:**
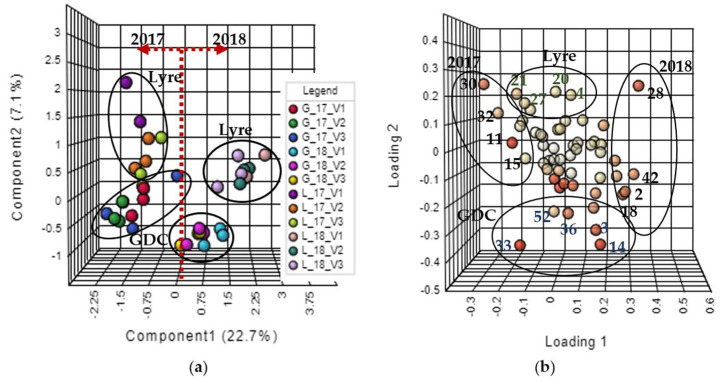
Partial least squares discriminant analysis plots. (**a**) Score plot of the two principal components of Chardonnay sparkling wines trained on lyre (L) and GDC (G) systems in two seasons (2017 and 2018) and a replicate of vinifications (V). (**b**) Loading plot of the two principal components.

The multivariate analysis was conducted with the normalised areas of 54 compounds presented in both systems and seasons (Table 1). Figure 1a shows the two main components of PLS-DA, which explains 29.8% of the data variability. The first component differentiates the vintage in 22.7% (right side: 2018; left side: 2017), and the second component discriminates the training systems in 7.1% (upper part: lyre; bottom part: GDC). Each data point colour in Figure 1a represents a vinification performed in triplicate for the training systems in each season. Figure 1b presents the loading plots, showing the compounds that most influence both components (light yellow to dark red data points, close to and distant from the origin).

#### 3.1.1. Harvest Discrimination

**Figure 2:**
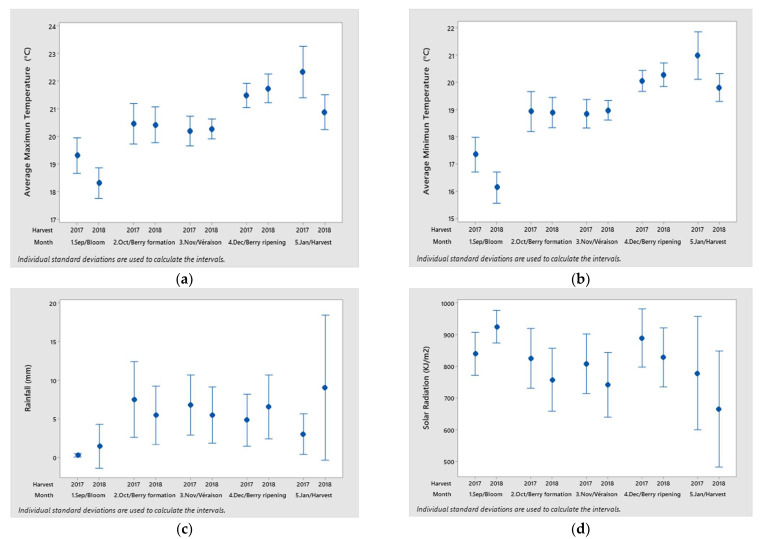
Environment conditions from bloom to harvest from two seasons (2017 and 2018). (**a**) Maximum temperature average (°C). (**b**) Minimum temperature average. (**c**) Rainfall (mm). (**d**) Solar radiation (KJ/m^2^).

Weather conditions significantly affect the compositions of grapes and wine, as shown in Figure 1a. Data concerning temperature, rainfall, and solar radiation between seasons (Figure 2) suggest that these factors may have contributed to variations in must content, biosynthesis of volatile compounds, and nutrient accumulation, which could have impacted the wine composition. Temperature can affect gene expression and enzyme activity, affecting primary and secondary metabolisms [22,23]. Radiation influences photosynthesis, secondary metabolism, and vine water status, impacting shoot development, berry ripening, and fruit composition [7].

Regarding the compounds separating the vintages (Figure 1b and Table 1), the right side is correlated with the 2018 vintage, with the following compounds: dodecanoic acid (ID28), nonanoic acid (ID42), isoamyl acetate (ID2), and hexyl acetate (ID18), while the compounds 2-ethylhexyl salicylate (ID11), acetaldehyde (ID15), ethyl-9-decenoate (ID30), and furfural (ID32) presented correlations with the 2017 vintage.

The 2017 vintage was differentiated by aldehydes (compounds related to wine oxidation); higher maximum and minimum temperatures at flowering and harvest stages; being a dry vintage at flowering, ripening, and harvest stages; and higher radiation at berry formation, *véraison*, ripening, and harvest stages.

The compound isoamyl acetate, correlated with the 2018 vintage, is related to the presence of the amino acid leucine, a precursor for the formation of isoamyl alcohol, which is subsequently esterified to isoamyl acetate through acetyltransferase enzyme action. It is an ester that can impact fruity aromas [24]. Additionally, this vintage is associated with lower temperatures, higher rainfall, and lower radiation—climatic conditions that could preserve the amino acids.

#### 3.1.2. Training System Differentiation

As shown in Figure 1a,b, the lyre system is differentiated at the top and the GDC at the bottom. The two systems evaluated have the vegetative canopy divided. However, the exposure or non-exposure of leaves and clusters to solar radiation and the microclimates established inside the canopies differ, influencing plant metabolism and the accumulation of primary and secondary metabolites in the berries.

The nutrients present in the must (sugars, lipids, amino acids, and acids) influence fermentation kinetics, as they affect yeast growth and fermentation speed [25] and can modulate the accumulation of certain compounds in sparkling wines, yielding different volatile profiles for each system.

The compounds that discriminate the lyre system are diethyl succinate (ID21), dimethyl sulfide (ID27), hexanol (ID4), and ethyl phenylacetate (ID20). For GDC, the discriminative compounds are geranylacetone (ID33), β-damascenone (ID52), isobutyl hexanoate (ID36), decanol (ID3), and 3-hexen-1-ol (E) (ID14), differentiated by alcohols, terpenoid compounds, and an ester.

The terpenoid compounds geranylacetone and β-damascenone differentiated the GDC system; these compounds can contribute fruity and floral aromas to sparkling wine with, which are considered positive for wine bouquet, being compounds biosynthesised mainly by the berries, suffering little or no interference during fermentation (hydrolysis of the glycosylated compounds). Additionally, the norisoprenoid compound β-damascenone, formed from the carotenoid neoxanthin, can alter the perception of other compounds [26]. Therefore, the multivariate analysis could indicate that the GDC system conferred more pleasant aromas to the sparkling wines.

The most relevant compound showed that the GDC system is more closely associated with the precursor linolenic acid due to the biosynthesis of 3-hexen-1-ol (E) and the lyre system with the precursor linoleic acid due to the formation of hexanol. This may indicate that the synthesis of a particular precursor is influenced by the conduction system. In work conducted by Xu et al. [27] in the northwest of China, the authors reported the influence of three training systems (a modified vertical shoot positioned trellis, a fan training system with two trunks, and a fan training system with multiple trunks) on fatty acid compositions and their volatile derivatives in grapes and wines, showing differences among them.

Some compounds were identified only in the sparkling wines (second fermentation and ageing on lees), dimethyl sulfide, furfural, nonanol, diethyl glutarate, ethyl-2-hydroxy-4-methylvalerate, ethyl valerate, ethyl lactate, and ethyl isobutyrate, and their presence was independent of the training system. The ethyl ester fatty acids present in sparkling wines present sweet and fruity aromas [28,29], and some of these esters (ethyl lactate and ethyl isovalerate) were identified in sparkling wines that spent 14 months in *sur lie* due to the presence of lees and the contact time with them [30]. One explanation is that reactions can occur and release fatty acids and derivatives with high odor activity [26,31]. Thus, it can be inferred that these compounds may have been formed during the ageing time (18 months).

The aromatic profiles of the sparkling wines, besides being differentiated by vintages and by training systems, showed that the accumulated nutrients present in the berries due to the metabolic processes occurring within the plants interfered with the kinetic reactions during fermentation, influencing the metabolism of yeast and consequently the *bouquets* of the sparkling wines.

## 4. Conclusions

This research concerns the volatile composition of sparkling wines produced in Serra da Mantiqueira (southeastern Brazil), a relatively new fine wine-producing region, harvested during the traditional grape period in the southern hemisphere (summer). With this evaluation, we sought to present information about the volatile profiles of these products; evaluate the compounds that grape berries can contribute to final products in tropical regions; increase knowledge of the varieties grown under these edaphoclimatic conditions—which is the novel contribution of this article; stimulate further studies concerning sparkling wines; and provide additional information to winegrowers managing vineyards to achieve products with different volatile compositions.

## Figures and Tables

**Table 1:** Compounds identified for both training systems and seasons in a replicate of vinifications of Chardonnay grapes harvested in the southeast of Brazil (Caldas, Minas Gerais), with the following features: compound identification (ID), loading of each compound for components 1 and 2, retention time (RT), modified Kovats retention indeces (mKRI) from the literature and obtained, and the identification approach.

Compound	ID	Component 1	Component 2	RT (min)	mKRI Literature	mKRI Obtained	Identification
Acetaldehyde	15	**−0.1534**	−0.0630	1.66	694	715	MS, RI
Dimethyl sulfide	27	−0.1279	**0.1737**	1.92	777	748	MS, RI
Ethyl acetate	31	−0.0647	0.0002	3.47	885	882	MS, RI
Ethanol	29	−0.0981	0.0518	5.09	926	955	MS, RI
Ethyl isobutyrate	51	0.0505	−0.0400	5.56	975	972	MS, RI
Ethyl butanoate	25	0.1784	−0.0247	7.77	1028	1045	MS, RI
Propanol	7	−0.0136	−0.1219	8.00	1030	1053	MS, RI
Ethyl 2-methylbutyrate	23	−0.0661	0.0495	8.26	1073	1061	MS, RI
Ethyl isovalerate	24	0.0812	−0.0071	8.74	1082	1075	MS, RI
2-Methyl-1-propanol	8	−0.0420	−0.1699	9.78	1077	1106	MS, RI
Isoamyl acetate	2	**0.2802**	−0.1777	10.33	1125	1126	MS, RI
Ethyl valerate	48	0.0461	−0.1925	10.71	1139	1140	MS, RI
Ethyl (E)-crotonate	10	0.2455	−0.1151	11.60	1158	1170	MS, RI
Methyl hexanoate	38	0.1782	0.0013	12.26	1184	1191	MS, RI
3-Methyl-1-butanol	1	−0.0166	−0.0551	13.14	1208	1224	MS, RI
Ethyl hexanoate	37	0.1082	0.1071	13.73	1223	1246	MS, RI
Styrene	53	0.1526	−0.2320	14.21	1241	1264	MS, RI
Hexyl acetate	18	**0.2723**	−0.2054	14.65	1276	1280	MS, RI
Ethyl heptanoate	34	−0.1611	0.0789	16.20	1328	1342	MS, RI
Ethyl lactate	50	−0.0214	−0.0121	16.48	1340	1354	MS, RI
Ethyl 2-hexenoate	13	0.2388	−0.0487	16.52	1336	1355	MS, RI
Isobutyl hexanoate	36	0.0316	**−0.2970**	16.69	1347	1362	MS, RI
Hexanol	4	0.0466	**0.2038**	16.78	1357	1365	MS, RI
3-Hexen-1-ol, (E)-	14	0.1744	**−0.4217**	17.02	1366	1375	MS, RI
3-Ethoxypropanol	9	−0.0172	−0.2072	17.38	1378	1389	MS, RI
Ethyl octanoate	45	0.0374	0.1040	18.86	1428	1454	MS, RI
Acetic acid	16	0.0578	−0.0068	18.99	1465	1460	MS, RI
Isopentyl hexanoate	39	−0.0299	−0.0768	19.20	1464	1469	MS, RI
Furfural	32	**−0.2576**	0.1309	19.30	1460	1474	MS, RI
Benzaldehyde	19	−0.0439	−0.0680	20.70	1508	1537	MS, RI
Ethyl 2-hydroxy-4-methylvalerate	47	0.0115	−0.0531	21.07	1515	1554	MS, RI
Octanol	6	−0.0644	0.0378	21.38	1546	1569	MS, RI
Ethyl furoate	12	−0.1084	0.1468	22.72	1621	1634	MS, RI
Butanoic acid	22	0.1521	−0.2137	22.83	1637	1639	MS, RI
Ethyl decanoate	26	0.0088	0.0715	23.04	1633	1650	MS, RI
Isoamyl octanoate	44	0.0213	0.0759	23.42	1658	1668	MS, RI
Nonanol	5	0.0048	−0.1859	23.49	1666	1672	MS, RI
Diethyl succinate	21	−0.1651	**0.2098**	23.81	1687	1688	MS, RI
Ethyl-9-decenoate	30	**−0.3067**	0.2488	24.08	1689	1700	MS, RI
α-Terpineol	54	0.1871	0.0733	24.31	1680	1713	MS, RI
Decanol	3	0.1540	**−0.3653**	25.46	1769	1774	MS, RI
Diethyl glutarate	46	−0.0574	−0.0677	25.80	1780	1791	MS, RI
Ethyl phenylacetate	20	−0.0174	**0.2171**	25.98	1779	1800	MS, RI
Phenethyl acetate	17	0.1777	−0.1241	26.56	1825	1833	MS, RI
β -Damascenone	52	−0.0145	**−0.2596**	26.73	1834	1842	MS, RI
Hexanoic acid	35	0.0555	−0.1095	26.96	1857	1855	MS, RI
Geranylacetone	33	−0.1935	**−0.4530**	27.20	1862	1868	MS, RI
Phenylethyl alcohol	49	−0.1523	0.1007	28.32	1912	1931	MS, RI
Octanoic acid	43	0.0814	0.0110	30.72	2070	2072	MS, RI
Nonanoic acid	42	**0.3173**	−0.1197	32.44	2169	2179	MS, RI
Hexyl salicylate	41	0.1472	0.0067	33.30	2203	2235	MS, RI
Decanoic acid	40	0.1389	0.0054	34.05	2281	2284	MS, RI
2-Ethylhexyl salicylate ^1^	11	**−0.1511**	0.0325	34.62	NF	2322	MS
Dodecanoic acid	28	**0.3337**	0.2406	37.18	2502	2500	MS, RI

NF: Not found; MS: compound identified by MS spectra (similarity ≥ 70%); RI: compound identified by comparing Kovats retention indices from the literature (accepted standard deviation of RI not exceeding 50 “Adapted with permission from Ref. [21]). 2022, N. Navrot”. mKRI: calculated by the temperature-programed Kovats index (logarithmic). ^1^ Based on the fragmentation profile similarity compared to the NIST library and score, 2-Ethylhexyl salicylate was tentatively identified. The compounds that showed higher loading for components 1 and 2 are in bold.

## Data Availability

Data is contained within the article.

## Supplementary Materials

The following supporting information can be downloaded at: https://www.mdpi.com/article/10.3390/foods11111529/s1. Figure S1: Expanded sample chromatogram with the main volatile compounds identified for the GDC system; Table S1: Normalised areas of the volatile compounds (area of the compound/area of the internal standard) for the GDC training system in the summer vintage of 2017 in Caldas, MG (Brazil) in replicate bottles and vinifications; Table S2: Normalised areas of the volatile compounds (area of the compound/area of the internal standard) for the GDC training system in the summer vintage of 2018 in Caldas, MG (Brazil), in replicate bottles and vinifications; Table S3: Normalised areas of the volatile compounds (area of the compound/area of the internal standard) for the lyre training system in the summer vintage of 2017 in Caldas, MG (Brazil) in replicate bottles and vinifications; Table S4: Normalised areas of the volatile compounds (area of the compound/area of the internal standard) for the lyre training system in the summer vintage of 2018 in Caldas, MG (Brazil) in replicate bottles and vinifications.
