# Supplementary material for: Volatile Composition of Sparkling Wines of cv. Chardonnay Cultivated under Different Training Systems in Serra da Mantiqueira (Brazil)
Source: Foods. 2022 May 24;11(11):1529. doi: 10.3390/foods11111529 (PMC9180589; doi:10.3390/foods11111529)
Supplement: Supplementary file 1 [file foods-11-01529-s001.zip › foods-1717322-supplementary.pdf]

Supplementary material:

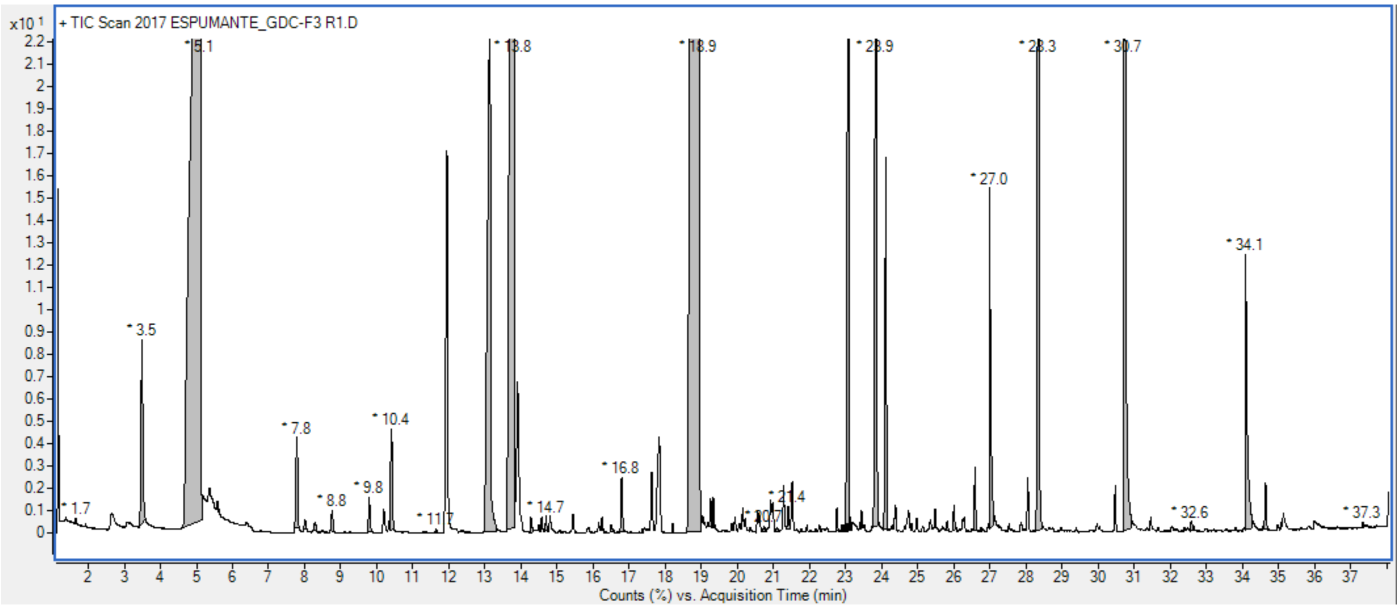

Figure S1. Expanded sample chromatogram with the main volatile compounds identified for the GDC system.

Table S1. Normalised areas of the volatile compounds (area of the compound/area of the internal standard) for the GDC training system in the summer vintage of 2017 in Caldas – MG (Brazil) in replicate bottles and vinifications.

GDC 2017			Vinification 1			Vinification 2			Vinification 3		
**Class**	**Compound**	**ID**	**B1**	**B2**	**B3**	**B1**	**B2**	**B3**	**B1**	**B2**	**B3**
Acids	Acetic acid	16	0.0087	0.0179	0.0188	0.0112	0.0219	0.0229	0.0206	0.0261	0.0260
	Butanoic acid	22	0.0070	0.0075	0.0071	0.0033	0.0055	0.0046	0.0083	0.0103	0.0074
	Dodecanoic acid	28	0.0059	00028	0.0040	0.0083	0.0000	0.0026	0.0134	0.0031	0.0052
	Hexanoic acid	35	0.4727	0.3857	0.3868	0.5187	0.4049	0.3820	0.6241	0.5820	0.4219
	Decanoic acid	40	0.5332	0.2527	0.4633	0.5450	0.2711	0.2700	0.6108	0.3047	0.3893
	Nonanoic acid	42	0.0372	0.0056	0.0110	0.0265	0.0093	0.0078	0.0277	0.0053	0.0092
	Octanoic acid	43	2.3384	1.6455	2.0263	2.5766	1.8770	1.6182	2.7376	2.1031	1.8687
Alcohols	3-Methyl-1-butanol	1	1.9028	1.6400	1.7379	1.9083	1.7594	1.6095	1.7533	1.8438	1.9140
	Decanol	3	0.0431	0.0111	0.0155	0.0431	0.0155	0.0419	0.0413	0.0125	0.0475
	Hexanol	4	0.1226	0.1141	0.1101	0.1356	0.1147	0.1142	0.1154	0.1027	0.0910
	Nonanol	5	0.0125	0.0071	0.0096	0.0109	0.0069	0.0116	0.0105	0.0059	0.0108
	Octanol	6	0.0406	0.0350	0.0394	0.0439	0.0334	0.0333	0.0496	0.0346	0.0342
	Propanol	7	0.0279	0.0300	0.0302	0.0296	0.0271	0.0302	0.0301	0.0366	0.0343
	2-Methyl-1-propanol	8	0.0795	0.0822	0.0848	0.0811	0.0645	0.0728	0.0790	0.0824	0.0746
	3-Hexen-1-ol, (*E*)-	14	0.0024	0.0019	0.0021	0.0024	0.0018	0.0017	0.0025	0.0022	0.0015
	Phenylethyl alcohol	49	2.1450	1.6559	1.7861	2.6425	1.9627	1.8849	2.0095	1.8651	1.3848
Alde hyde	Acetaldehyde	15	0.0063	0.0055	0.0064	0.0094	0.0062	0.0079	0.0075	0.0092	0.0061
	Benzaldehyde	19	0.0080	0.0087	0.0062	0.0090	0.0059	0.0074	0.0106	0.0130	0.0069
	Furfural	32	0.0649	0.516	0.0566	0.0706	0.0614	0.0591	0.0559	0.0617	0.0568
Esters	Ethyl (E)-crotonate	10	0.0056	0.0044	0.0041	0.0065	0.0035	0.0032	0.0083	0.0023	0.0055
	2-Ethylhexyl salicylate	11	0.0497	0.0016	0.0032	0.0568	0.0022	0.0023	0.0917	0.0019	0.0038
	Ethyl furoate	12	0.0449	0.0347	0.0342	0.0516	0.0311	0.0319	0.0426	0.0327	0.0321
	Ethyl 2-hexenoate	13	0.0106	0.0042	0.0041	0.0074	0.0069	0.0110	0.0073	0.0000	0.0000
	Ethyl phenylacetate	20	0.0478	0.0431	0.0440	0.0559	0.0437	0.0408	0.0465	0.0296	0.0315
	Diethyl succinate	21	2.6070	2.3962	2.5393	3.2614	2.5555	2.5524	2.3344	2.5264	1.8489
	Ethyl 2-methylbutyrate	23	0.0432	0.0474	0.0478	0.0487	0.0468	0.0433	0.0262	0.0031	0.0289

	Ethyl isovalerate	24	0.0617	0.0642	0.0665	0.0650	0.0642	00597	0.0520	0.0071	0.0545
	Ethyl butanoate	25	0.2240	0.2078	0.2104	0.2279	0.2022	0.1915	0.2498	0.0689	0.2394
	Ethyl decanoate	26	1.4272	1.0268	1.1623	1.5649	0.8137	0.7929	1.7057	1.5591	1.4124
	Ethyl 9-decenoate	30	0.7623	0.6446	0.7517	0.9100	0.6036	0.5845	0.7132	0.1415	0.5943
	Ethyl heptanoate	34	0.0274	0.0269	0.0271	0.0382	0.0525	0.0295	0.0316	0.0051	0.0291
	Isobutyl hexanoate	36	0.0018	0.0024	0.0027	0.0025	0.0031	0.0024	0.0029	0.0000	0.0033
	Ethyl hexanoate	37	4.6643	4.2583	4.3180	5.0140	4.0265	3.8205	6.2573	5.7173	5.1772
	Methyl hexanoate	38	0.0031	0.0027	0.0027	0.0038	0.0000	0.0000	0.0043	0.0000	0.0000
	Isopentyl hexanoate	39	0.0363	0.0313	0.0310	0.0447	0.0424	0.0382	0.0476	0.0140	0.0415
	Isoamyl octanoate	44	0.0372	0.0305	0.0285	0.0428	0.0206	0.0269	0.0457	0.0000	0.0355
	Ethyl octanoate	45	14.6251	12.3989	13.0691	16.4038	11.3192	11.0516	19.2129	17.4126	15.6122
	Diethyl glutarate	46	0.0141	0.0103	0.0118	0.0175	0.0116	0.0101	0.0165	0.0137	0.0103
	Ethyl 2-hydroxy-4-methylvalerate	47	0.0084	0.0101	0.0103	0.0089	0.0087	0.0067	0.0089	0.0120	0.0080
	Ethyl valerate	48	0.0040	0.0029	0.0049	0.0024	0.0044	0.0032	0.0023	0.0000	0.0028
	Ethyl lactate	50	0.0171	0.0208	0.0195	0.0242	0.0180	0.0138	0.0152	0.0212	0.0195
	Ethyl isobutyrate	51	0.0362	0.0330	0.0373	0.0303	0.0199	0.0305	0.0264	0.0119	0.0216
	Isoamyl acetate	2	0.1762	0.1384	0.1297	0.1739	0.1191	0.1075	0.2853	0.0266	0.1743
	Phenethyl acetate	17	0.0826	0.0632	0.0597	0.0813	0.0606	0.0498	0.1158	0.0694	0.0715
	Hexyl acetate	18	0.0201	0.0115	0.0103	0.0160	0.0171	0.0091	0.0283	0.0000	0.0179
	Ethyl acetate	31	0.4392	0.4186	0.4234	0.4445	0.3923	0.3973	0.5202	0.3849	0.5085
**Ether**	3-Ethoxypropanol	9	0.0033	0.0033	0.0037	0.0042	0.0026	0.0025	0.0065	0.0046	0.0040
**H**	Styrene	53	0.0242	0.0209	0.0226	0.0348	0.0242	0.0171	0.0334	0.0089	0.0306
**C13-NP**	β -Damascenone	52	0.0115	0.0121	0.0121	0.0224	0.0090	0.0066	0.0052	0.0090	0.0126
**Phenol**	Hexyl salicylate	41	0.0000	0.0016	0.0015	0.0000	0.0023	0.0015	0.0000	0.0000	0.0017
**Sulfide**	Dimethyl sulfide	27	0.0035	0.0016	0.0012	0.0037	0.0010	0.0010	0.0051	0.0009	0.0014
**T**	Geranylacetone	33	0.0253	0.0129	0.0324	0.0174	0.0567	0.0472	0.0000	0.0239	0.0441
	α-Terpineol	54	0.0000	0.0072	0.0059	0.0000	0.0048	0.0018	0.0000	0.0061	0.0034
**EtOH**	Ethanol	29	9.2551	9.0079	9.1884	9.5155	8.9934	9.0283	9.4441	9.5748	10.4312

ID: compounds identification; B: bottle; H: hydrocarbon; C13-NP: C13-norisoprenoid; T: terpenoid; EtOH: ethanol

Table S2. Normalised areas of the volatile compounds (area of the compound/area of the internal standard) for the GDC training system in the summer vintage of 2018 in Caldas – MG (Brazil) in replicate bottles and vinifications.

GDC 2018			Vinification 1			Vinification 2			Vinification 3		
**Class**	**Compound**	**ID**	**B1**	**B2**	**B3**	**B1**	**B2**	**B3**	**B1**	**B2**	**B3**
**Acids**	Acetic acid	16	0.0366	0.0240	0.0323	0.0296	0.0241	0.0215	0.0376	0.0226	0.0317
	Butanoic acid	22	0.0135	0.0155	0.0143	0.0127	0.0133	0.0131	0.0140	0.0154	0.0149
	Dodecanoic acid	28	0.0120	0.0093	0.0187	0.0101	0.0030	0.0068	0.0034	0.0057	0.0022
	Hexanoic acid	35	0.4521	0.5102	0.6016	0.5036	0.4688	0.4997	0.4838	0.5551	0.5271
	Decanoic acid	40	0.6095	0.4826	0.5319	0.8093	0.3195	0.6186	0.3501	0.5872	0.4994
	Nonanoic acid	42	0.0419	0.0577	0.0708	0.0101	0.0046	0.0081	0.0053	0.0073	0.0067
	Octanoic acid	43	2.3347	2.2474	2.7820	2.8193	2.1047	2.4049	2.2758	2.5282	2.6611
**Alcohols**	3-Methyl-1-butanol	1	2.1042	2.1676	2.1453	2.1264	2.1443	1.9745	2.1988	2.1211	2.1121
	Decanol	3	0.0332	0.0324	0.0353	0.0417	0.0226	0.0390	0.0282	0.0417	0.0372
	Hexanol	4	0.0955	0.1015	0.1176	0.0983	0.1046	0.0998	0.1020	0.1065	0.1024
	Nonanol	5	0.0072	0.0086	0.0061	0.0091	0.0059	0.0107	0.0103	0.0104	0.0126
	Octanol	6	0.0293	0.0358	0.0384	0.0355	0.0380	0.0398	0.0267	0.0365	0.0294
	Propanol	7	0.0275	0.0292	0.0261	0.0288	0.0315	0.0293	0.0277	0.0280	0.0261
	2-Methyl-1-propanol	8	0.0785	0.0655	0.0000	0.0781	0.0892	0.0790	0.0911	0.0808	0.0804
	3-Hexen-1-ol, (*E*)-	14	0.0016	0.0021	0.0035	0.0021	0.0024	0.0021	0.0021	0.0018	0.0022
	Phenylethyl alcohol	49	1.2426	1.3824	1.6570	1.5962	1.3076	1.4619	1.5119	1.6808	1.6754
**Alde hyde**	Acetaldehyde	15	0.0062	0.0111	0.0050	0.0066	0.0051	0.0041	0.0043	0.0054	0.0099
	Benzaldehyde	19	0.0063	0.0090	0.0050	0.0052	0.0063	0.0038	0.0058	0.0068	0.0077
	Furfural	32	0.0290	0.0524	0.0399	0.0270	0.0335	0.0255	0.0308	0.0287	0.0501
**Esters**	Ethyl (E)-crotonate	10	0.0096	0.0088	0.0131	0.0080	0.0084	0.0082	0.0086	0.0077	0.0069
	2-Ethylhexyl salicylate	11	0.0015	0.0015	0.0020	0.0044	0.0026	0.0046	0.0039	0.0031	0.0043
	Ethyl furoate	12	0.0287	0.0325	0.0351	0.0279	0.0311	0.0296	0.0276	0.0276	0.0303
	Ethyl 2-hexenoate	13	0.0083	0.0073	0.0092	0.0046	0.0150	0.0112	0.0071	0.0093	0.0103
	Ethyl phenylacetate	20	0.0371	0.0378	0.0438	0.0411	0.0401	0.0395	0.0436	0.0439	0.0411
	Diethyl succinate	21	1.0614	1.1839	1.3920	1.2399	1.1676	1.1957	1.1795	1.2750	1.2654
	Ethyl 2-methylbutyrate	23	0.0294	0.0316	0.0342	0.0359	0.0391	0.0384	0.0372	0.0365	0.0365
	Ethyl isovalerate	24	0.0737	0.0767	0.0830	0.0757	0.0828	0.0795	0.0838	0.0826	0.0768
	Ethyl butanoate	25	0.2898	0.3166	0.3314	0.2994	0.3224	0.3113	0.3120	0.3154	0.2938
	Ethyl decanoate	26	1.4188	0.9067	1.1562	1.5754	1.0731	0.8948	1.4820	0.9558	1.1504
	Ethyl 9-decenoate	30	0.1889	0.1237	0.1548	0.2252	0.1677	0.1445	0.2115	0.1419	0.1587
	Ethyl heptanoate	34	0.0178	0.0172	0.0176	0.0194	0.0213	0.0179	0.0175	0.0172	0.0160
	Isobutyl hexanoate	36	0.0030	0.0030	0.0042	0.0025	0.0031	0.0027	0.0018	0.0029	0.0030
	Ethyl hexanoate	37	4.8333	5.1785	5.8339	4.7797	5.0253	4.8930	5.1003	5.0953	4.6452
	Methyl hexanoate	38	0.0047	0.0034	0.0000	0.0017	0.0043	0.0039	0.0041	0.0022	0.0036
	Isopentyl hexanoate	39	0.0469	0.0443	0.0560	0.0473	0.0427	0.0385	0.0502	0.0394	0.0421
	Isoamyl octanoate	44	0.0315	0.0256	0.0266	0.0339	0.0252	0.0246	0.0414	0.0343	0.0333
	Ethyl octanoate	45	14.0262	13.1191	14.9477	14.5616	12.7034	11.9241	14.7326	12.1270	12.7288
	Diethyl glutarate	46	0.0083	0.0082	0.0105	0.0099	0.0078	0.0079	0.0083	0.0089	0.0111
	Ethyl 2-hydroxy-4-methylvalerate	47	0.01000	0.0130	0.0127	0.0129	0.0123	0.0110	0.0108	0.0120	0.0107
	Ethyl valerate	48	0.0014	0.0033	0.0031	0.0025	0.0048	0.0033	0.0017	0.0028	0.0023
	Ethyl lactate	50	0.0189	0.0325	0.0279	0.0282	0.0309	0.0303	0.0191	0.0183	0.0143
	Ethyl isobutyrate	51	0.0238	0.0235	0.0375	0.0364	0.0378	0.0332	0.0328	0.0398	0.0365
	Isoamyl acetate	2	0.3701	0.3525	0.3009	0.3044	0.3262	0.3299	0.3803	0.3512	0.3241
	Phenethyl acetate	17	0.1191	0.1032	0.1543	0.1166	0.0946	0.1045	0.1389	0.1290	0.1254
	Hexyl acetate	18	0.0248	0.0243	0.0378	0.0201	0.0208	0.0213	0.0255	0.0268	0.0204
	Ethyl acetate	31	0.4118	0.4197	0.4149	0.4085	0.4414	0.4147	0.4482	0.4422	0.4149
**Ether**	3-Ethoxypropanol	9	0.0033	0.0039	0.0000	0.0034	0.0043	0.0041	0.0026	0.0039	0.0036
**H**	Styrene	53	0.0566	0.0615	0.0778	0.0638	0.0510	0.0516	0.0666	0.0538	0.0586
**C13-NP**	β-Damascenone	52	0.0168	0.0397	0.0191	0.0173	0.0211	0.0159	0.0111	0.0132	0.0405
**Phenol**	Hexyl salicylate	41	0.0014	0.0012	0.0022	0.0031	0.0018	0.0020	0.0026	0.0018	0.0031
**Sulfide**	Dimethyl sulfide	27	0.0010	0.0011	0.0009	0.0020	0.0010	0.0014	0.0008	0.0014	0.0014

T	Geranylacetone	33	0.0173	0.0176	0.0160	0.0215	0.0204	0.0299	0.0284	0.0357	0.0199
	α-Terpineol	54	0.0044	0.0075	0.0046	0.0066	0.0050	0.0053	0.0055	0.0047	0.0044
EtOH	Ethanol	29	7.4940	8.1692	10.5290	8.2099	8.3423	7.9751	7.9963	8.5473	8.0469

ID: compounds identification; B: bottle; H: hydrocarbon; C13-NP: C13-norisoprenoid; T: terpenoid; EtOH: ethanol

Table S3. Normalised areas of the volatile compounds (area of the compound/area of the internal standard) for the lyre training system in the summer vintage of 2017 in Caldas – MG (Brazil) in replicate bottles and vinifications.

	**Lyre 2017**		**Vinification 1**			**Vinification 2**			**Vinification 3**		
**Class**	**Compound**	**ID**	**B1**	**B2**	**B3**	**B1**	**B2**	**B3**	**B1**	**B2**	**B3**
**Acids**	Acetic acid	16	0.0548	0.0388	0.0301	0.0134	0.0261	0.0214	0.0093	0.0162	0.0193
	Butanoic acid	22	0.0157	0.0043	0.0051	0.0067	0.0096	0.0069	0.0062	0.0085	0.0100
	Dodecanoic acid	28	0.0000	0.0057	0.0045	0.0060	0.0064	0.0049	0.0079	0.0030	0.0036
	Hexanoic acid	35	0.6816	0.5050	0.4042	0.5348	0.5151	0.4226	0.4897	0.3906	0.0375
	Decanoic acid	40	0.4295	0.3415	0.3363	0.5339	0.4369	0.3799	0.6098	0.4045	0.3813
	Nonanoic acid	42	0.0065	0.0000	0.0000	0.0113	0.0114	0.0054	0.0410	0.0081	0.0084
	Octanoic acid	43	2.7215	2.2846	1.9343	2.3202	2.4885	2.0297	2.4450	1.7760	1.7697
**Alcohols**	3-Methyl-1-butanol	1	1.9097	2.3016	1.6411	1.7662	1.7258	1.7115	1.9305	1.9168	1.9759
	Decanol	3	0.0532	0.0000	0.0000	0.0386	0.0423	0.0388	0.0198	0.0121	0.0151
	Hexanol	4	0.1607	0.1314	0.1118	0.1427	0.1158	0.1128	0.1311	0.1214	0.1244
	Nonanol	5	0.0156	0.0000	0.0104	0.0099	0.0125	0.0060	0.0103	0.0167	0.0118
	Octanol	6	0.0487	0.0357	0.0307	0.0461	0.0331	0.0341	0.0402	0.0380	0.0350
	Propanol	7	0.0334	0.0115	0.0285	0.0294	0.0292	0.0296	0.0254	0.0273	0.0294
	2-Methyl-1-propanol	8	0.0814	0.0000	0.0808	0.0797	0.0483	0.0681	0.0758	0.0821	0.0841
	3-Hexen-1-ol, (*E*)-	14	0.0016	0.0000	0.0000	0.0010	0.0008	0.0012	0.0010	0.0010	0.0012
	Phenylethyl alcohol	49	2.4078	2.0420	1.6873	2.3480	2.4085	1.9734	2.3982	1.8482	1.9093
**Alde hyde**	Acetaldehyde	15	0.0134	0.0042	0.0061	0.0095	0.0081	0.0070	0.0074	0.0081	0.0092
	Benzaldehyde	19	0.0066	0.0038	0.0060	0.0083	0.0079	0.0044	0.0073	0.0053	0.0045
	Furfural	32	0.0894	0.0733	0.0642	0.0782	0.0649	0.0613	0.0755	0.0598	0.0612
**Esters**	Ethyl (E)-crotonate	10	0.0066	0.0040	0.0044	0.0055	0.0040	0.0039	0.0053	0.0037	0.0038
	2-Ethylhexyl salicylate	11	0.0201	0.0030	0.0000	0.1536	0.0022	0.0029	0.0643	0.0033	0.0043
	Ethyl furoate	12	0.0474	0.0511	0.0326	0.0481	0.0327	0.0354	0.0434	0.0319	0.0330
	Ethyl 2-hexenoate	13	0.0076	0.0051	0.0047	0.0079	0.0000	0.0000	0.0059	0.0053	0.0037
	Ethyl phenylacetate	20	0.0543	0.0470	0.0490	0.0598	0.0557	0.0555	0.0609	0.0505	0.0493
	Diethyl succinate	21	0.3078	2.7776	2.4335	2.7959	2.9450	2.6634	2.8923	2.5416	2.6625
	Ethyl 2-methylbutyrate	23	0.0450	0.0361	0.0341	0.0423	0.0410	0.0418	0.0438	0.0488	0.0510
	Ethyl isovalerate	24	0.0785	0.0670	0.0577	0.0661	0.0627	0.0637	0.0663	0.0688	0.0743
	Ethyl butanoate	25	0.2873	0.2272	0.2095	0.2545	0.2205	0.2165	0.2321	0.2165	0.2294
	Ethyl decanoate	26	2.6588	1.2146	1.1478	1.6091	1.0520	1.2606	1.3193	0.7463	1.0461
	Ethyl 9-decenoate	30	0.9993	0.5423	0.6011	0.7118	0.5303	0.6839	0.6223	0.3951	0.5185
	Ethyl heptanoate	34	0.0413	0.0196	0.0239	0.0318	0.0339	0.0299	0.0262	0.0277	0.0276
	Isobutyl hexanoate	36	0.0043	0.0000	0.0026	0.0018	0.0025	0.0029	0.0021	0.0029	0.0025
	Ethyl hexanoate	37	7.0331	5.2088	4.3755	5.8505	4.5438	4.4664	4.7390	4.3576	4.4428
	Methyl hexanoate	38	0.0055	0.0000	0.0029	0.0039	0.0000	0.0011	0.0032	0.0030	0.0031
	Isopentyl hexanoate	39	0.0641	0.0536	0.0364	0.0401	0.0354	0.0345	0.0388	0.0313	0.0362
	Isoamyl octanoate	44	0.0860	0.0543	0.0302	0.0449	0.0355	0.0239	0.0000	0.0312	0.0295
	Ethyl octanoate	45	23.1113	13.7033	13.4469	16.9651	12.3022	13.9949	14.6017	10.9568	12.4539
	Diethyl glutarate	46	0.0143	0.0110	0.0000	0.0129	0.0146	0.0115	0.0131	0.0108	0.0106
	Ethyl 2-hydroxy-4-methylvalerate	47	0.0072	0.0116	0.0091	0.0092	0.0097	0.0073	0.0088	0.0105	0.0094
	Ethyl valerate	48	0.0063	0.0000	0.0025	0.0022	0.0027	0.0033	0.0018	0.0036	0.0050
	Ethyl lactate	50	0.0252	0.0240	0.0182	0.0228	0.0281	0.0252	0.0234	0.0208	0.0239
	Ethyl isobutyrate	51	0.0406	0.0235	0.0186	0.0367	0.0241	0.0291	0.0310	0.0289	0.0384
	Isoamyl acetate	2	0.2160	0.0895	0.1273	0.1951	0.1225	0.1059	0.1610	0.1244	0.1122
	Phenethyl acetate	17	0.0778	0.0700	0.0597	0.0861	0.0735	0.0638	0.0842	0.0588	0.0513
	Hexyl acetate	18	0.0229	0.0000	0.0092	0.0256	0.0150	0.0094	0.0191	0.0103	0.0096

Class	Compound	ID	B1	B2	B3	B1	B2	B3	B1	B2	B3
	Ethyl acetate	31	0.5567	0.4123	0.4337	0.5057	0.4088	0.4177	0.4194	0.4018	0.4064
**Ether**	3-Ethoxypropanol	9	0.0065	0.0000	0.0017	0.0057	0.0046	0.0025	0.0016	0.0024	0.0030
**H**	Styrene	53	0.0429	0.0274	0.0236	0.0414	0.0250	0.0358	0.0307	0.0215	0.0213
**C13-NP**	β-Damascenone	52	0.0190	0.0117	0.0101	0.0115	0.0098	0.0104	0.0118	0.0105	0.0190
**Phenol**	Hexyl salicylate	41	0.0000	0.0026	0.0017	0.0000	0.0029	0.0027	0.0000	0.0016	0.0024
**Sulfide**	Dimethyl sulfide	27	0.0094	0.0022	0.0014	0.0058	0.0013	0.0014	0.0032	0.0012	0.0010
**T**	Geranylacetone	33	0.0133	0.0205	0.0615	0.0000	0.0167	0.0550	0.0238	0.0585	0.0005
	α-Terpineol	54	0.0000	0.0203	0.0039	0.0000	0.0071	0.0054	0.0000	0.0054	0.0040
**EtOH**	Ethanol	29	11.8132	10.4770	8.7179	10.1860	9.6305	9.4350	8.9473	8.9854	9.3999

ID: compounds identification; B: bottle; H: hydrocarbon; C13-NP: C13-norisoprenoid; T: terpenoid; EtOH: ethanol

Table S4. Normalised areas of the volatile compounds (area of the compound/area of the internal standard) for the lyre training system in the summer vintage of 2018 in Caldas – MG (Brazil) in replicate bottles and vinifications.

	**Lyre 2018**		**Vinification 1**			**Vinification 2**			**Vinification 3**		
**Class**	**Compound**	**ID**	**B1**	**B2**	**B3**	**B1**	**B2**	**B3**	**B1**	**B2**	**B3**
**Acids**	Acetic acid	16	0.0302	0.0237	0.0220	0.0197	0.0322	0.0255	0.0278	0.0292	0.0294
	Butanoic acid	22	0.0088	0.0114	0.0126	0.0084	0.0112	0.0135	0.0149	0.0124	0.0098
	Dodecanoic acid	28	0.0164	0.0806	0.0306	0.0149	0.0201	0.0202	0.0162	0.0156	0.0156
	Hexanoic acid	35	0.5968	0.5785	0.6400	0.5039	0.6473	0.5722	0.5996	0.5138	0.5908
	Decanoic acid	40	0.6327	0.7265	0.8602	0.6473	0.7654	0.6855	0.5817	0.3724	0.3020
	Nonanoic acid	42	0.0970	0.1050	0.0771	0.0316	0.0362	0.0440	0.0580	0.0514	0.0625
	Octanoic acid	43	2.6257	2.8048	3.2188	2.4337	3.0250	2.7724	3.1309	2.1234	2.6287
**Alcohols**	3-Methyl-1-butanol	1	1.7367	1.6197	1.8232	1.5976	2.3158	1.8218	1.7150	1.7710	1.6918
	Decanol	3	0.0398	0.0504	0.0562	0.0367	0.0462	0.0454	0.0351	0.0299	0.0419
	Hexanol	4	0.1572	0.1623	0.1617	0.1463	0.1695	0.1600	0.1471	0.1477	0.1522
	Nonanol	5	0.0100	0.0126	0.0123	0.0090	0.0135	0.0105	0.0094	0.0052	0.0049
	Octanol	6	0.0384	0.0425	0.0354	0.0297	0.0323	0.0352	0.0279	0.0304	0.0335
	Propanol	7	0.0261	0.0276	0.0306	0.0277	0.0404	0.0342	0.0286	0.0275	0.0242
	2-Methyl-1-propanol	8	0.0624	0.0703	0.0630	0.0634	0.0805	0.0697	0.0614	0.0585	0.0594
	3-Hexen-1-ol, (*E*)-	14	0.0023	0.0028	0.0027	0.0026	0.0034	0.0035	0.0025	0.0026	0.0038
	Phenylethyl alcohol	49	1.5097	1.3878	1.8036	1.2154	1.5987	1.4191	1.5523	1.1744	1.4018
**Alde hyde**	Acetaldehyde	15	0.0057	0.0045	0.0057	0.0048	0.0066	0.0040	0.0042	0.0041	0.0056
	Benzaldehyde	19	0.0058	0.0065	0.0075	0.0061	0.0069	0.0067	0.0081	0.0056	0.0094
	Furfural	32	0.0213	0.0239	0.0297	0.0211	0.0279	0.0270	0.0229	0.0572	0.0366
**Esters**	Ethyl (E)-crotonate	10	0.0093	0.0103	0.0093	0.0085	0.0103	0.0107	0.0083	0.0093	0.0089
	2-Ethylhexyl salicylate	11	0.0034	0.0026	0.0032	0.0022	0.0025	0.0027	0.0022	0.0025	0.0014
	Ethyl furoate	12	0.0306	0.0324	0.0336	0.0284	0.0341	0.0336	0.0296	0.0288	0.0311
	Ethyl 2-hexenoate	13	0.0113	0.0097	0.0088	0.0127	0.0149	0.0183	0.0107	0.0135	0.0168
	Ethyl phenylacetate	20	0.0465	0.0520	0.0548	0.0412	0.0518	0.0497	0.0544	0.0453	0.0496
	Diethyl succinate	21	1.3245	1.3299	1.4733	1.1092	1.3741	1.2620	1.3208	1.1039	1.2780
	Ethyl 2-methylbutyrate	23	0.0275	0.0267	0.0262	0.0234	0.0306	0.0280	0.0252	0.0263	0.0272
	Ethyl isovalerate	24	0.0672	0.0626	0.0638	0.0576	0.0744	0.0698	0.0589	0.0618	0.0628
	Ethyl butanoate	25	0.3190	0.3026	0.3178	0.2996	0.3758	0.3443	0.2939	0.3129	0.3081
	Ethyl decanoate	26	1.0698	0.8415	0.9756	1.1286	1.6620	1.0085	1.5858	0.9176	1.0582
	Ethyl 9-decenoate	30	0.1649	0.1321	0.1318	0.1520	0.2142	0.1680	0.2622	0.1907	0.1946
	Ethyl heptanoate	34	0.0167	0.0167	0.0171	0.0137	0.0180	0.0167	0.0142	0.0160	0.0339
	Isobutyl hexanoate	36	0.0018	0.0015	0.0022	0.0015	0.0030	0.0021	0.0019	0.0020	0.0032
	Ethyl hexanoate	37	5.3644	5.2096	5.1249	5.0459	6.0188	5.7996	4.9442	5.2395	5.3121
	Methyl hexanoate	38	0.0036	0.0044	0.0026	0.0042	0.0047	0.0045	0.0033	0.0032	0.0000
	Isopentyl hexanoate	39	0.0321	0.0343	0.0271	0.0332	0.0536	0.0384	0.0407	0.0080	0.0533
	Isoamyl octanoate	44	0.0258	0.0232	0.0325	0.0252	0.0476	0.0304	0.0373	0.0175	0.0202
	Ethyl octanoate	45	13.4723	12.0671	12.2883	12.8809	16.3360	13.9462	14.7012	13.9100	13.9177
	Diethyl glutarate	46	0.0090	0.0110	0.0122	0.0089	0.0116	0.0095	0.0123	0.0072	0.0095

	Ethyl 2-hydroxy-4-methylvalerate	47	0.0096	0.0107	0.0121	0.0069	0.0089	0.0095	0.0094	0.0095	0.0058
	Ethyl valerate	48	0.0053	0.0011	0.0026	0.0028	0.0041	0.0040	0.0021	0.0041	0.0024
	Ethyl lactate	50	0.0220	0.0209	0.0235	0.0161	0.0237	0.0158	0.0214	0.0189	0.0170
	Ethyl isobutyrate	51	0.0323	0.0287	0.0250	0.0275	0.0366	0.0362	0.0322	0.0303	0.0304
	Isoamyl acetate	2	0.3578	0.3464	0.3377	0.3218	0.3745	0.3688	0.3068	0.3146	0.2392
	Phenethyl acetate	17	0.1154	0.1227	0.1337	0.0977	0.1255	0.1190	0.1278	0.1006	0.1122
	Hexyl acetate	18	0.0340	0.0338	0.0319	0.0301	0.0343	0.0362	0.0322	0.0329	0.0376
	Ethyl acetate	31	0.4038	0.3965	0.4152	0.3556	0.4550	0.3976	0.3661	0.3817	0.3518
**Ether**	3-Ethoxypropanol	9	0.0028	0.0030	0.0035	0.0027	0.0050	0.0030	0.0032	0.0029	0.0032
**H**	Styrene	53	0.0343	0.0309	0.0352	0.0319	0.0426	0.0393	0.0320	0.0355	0.0397
**C13-NP**	β-Damascenone	52	0.0163	0.0110	0.0112	0.0075	0.0101	0.0075	0.0111	0.0079	0.0088
**Phenol**	Hexyl salicylate	41	0.0028	0.0014	0.0015	0.0015	0.0018	0.0019	0.0019	0.0015	0.0021
**Sulfide**	Dimethyl sulfide	27	0.0011	0.0014	0.0009	0.0010	0.0013	0.0021	0.0013	0.0016	0.0015
**T**	Geranylacetone	33	0.0043	0.0016	0.0034	0.0037	0.0038	0.0040	0.0057	0.0128	0.0195
	α-Terpineol	54	0.0072	0.0072	0.0091	0.0051	0.0079	0.0070	0.0082	0.0080	0.0094
**EtOH**	Ethanol	29	7.5826	7.4958	8.8089	7.3228	10.2925	8.3781	7.4779	7.5605	7.6726

ID: compounds identification; B: bottle; H: hydrocarbon; C13-NP: C13-norisoprenoid; T: terpenoid; EtOH: ethanol
